# Integration of real-world clinical data into the Munich Mental Health Biobank – clinical and scientific potential and challenges

**DOI:** 10.1192/j.eurpsy.2022.1455

**Published:** 2022-09-01

**Authors:** J. Kálmán, G. Burkhardt, O. Pogarell, F. Padberg, T. Schulze, P. Falkai

**Affiliations:** 1 University Hospital, LMU Munich, Department Of Psychiatry And Psychotherapy, Munich, Germany; 2 University Hospital, LMU Munich, Institute Of Psychiatric Phenomics And Genomics, Munich, Germany

**Keywords:** digitalization, biobank, real-world data, affective disorders

## Abstract

**Introduction:**

New insights into the pathophysiology of mental disorders and innovations in psychiatric care depend on the availability of representative, longitudinal and multidimensional datasets across diverse, transdiagnostic populations. Biobanks usually attempt to collect such data in parallel to clinical routine, which is resource-intensive, puts additional burden on health-care providers, and may reduce the generalizability of the results. Despite containing rich phenotypic and biological information, data generated in routine clinical care is seldomly used for research purposes, because it is usually unstructured and locked in data silos. To truly link clinical practice and research, solutions that optimize the generation and scientific utilization of real-world clinical data are needed.

**Objectives:**

Evaluation of a new digital infrastructure which warrants the efficient, automatized, and structured collection of real-world data in psychiatric care, and integrates the generated data into existing biobanking efforts.

**Methods:**

We have developed a new documentation system which augments the existing IT-structures, enables the collection of routine clinical data in a structured format and involves patients in the data generation process. In an implementation science approach, to replicate and extend the findings of Blitz et al. (JMIR Ment Health 2021), we are investigating the acceptance, efficacy, and safety of the system in our outpatient clinic for affective disorders.

**Results:**

First results describing the technical safety, usage metrics, and acceptance of the system, and the quality of the collected data will be presented.

**Conclusions:**

Challenges of collecting real-world data for biobanking and research purposes and perspectives on future digital solutions will be discussed.

**Disclosure:**

No significant relationships.

